# Investigating the anticancer activity of eravacycline in pancreatic cancer via target-based deep learning and experimental validation

**DOI:** 10.1093/bib/bbag353

**Published:** 2026-07-08

**Authors:** Adi Jabarin, Guy Shtar, Valeria Feinshtein, Eyal Mazuz, Bracha Shapira, Lior Rokach, Shimon Ben-Shabat

**Affiliations:** Department of Clinical Biochemistry and Pharmacology, Ben-Gurion University of the Negev, P.O.B. 653, Beer-Sheva 8410501, Israel; Department of Information Systems and Software Engineering, Ben-Gurion University of the Negev, P.O.B. 653, Beer-Sheva 8410501, Israel; Department of Clinical Biochemistry and Pharmacology, Ben-Gurion University of the Negev, P.O.B. 653, Beer-Sheva 8410501, Israel; Department of Information Systems and Software Engineering, Ben-Gurion University of the Negev, P.O.B. 653, Beer-Sheva 8410501, Israel; Department of Information Systems and Software Engineering, Ben-Gurion University of the Negev, P.O.B. 653, Beer-Sheva 8410501, Israel; Department of Information Systems and Software Engineering, Ben-Gurion University of the Negev, P.O.B. 653, Beer-Sheva 8410501, Israel; Department of Clinical Biochemistry and Pharmacology, Ben-Gurion University of the Negev, P.O.B. 653, Beer-Sheva 8410501, Israel

**Keywords:** eravacycline, molecular-based deep learning model, p53, POLK, pancreatic cancer

## Abstract

Pancreatic ductal adenocarcinoma (PDAC) is a highly lethal malignancy with limited therapeutic options. In this study, we introduce a target-based deep learning framework to investigate the anticancer activity of eravacycline (Erav), a United States Food and Drug Administration (FDA)-approved antibacterial agent previously identified in our work as a potential anticancer candidate through computational screening. We developed a novel two-phase *in silico* yeast-based prediction model to explore potential mechanisms of action, followed by *in vitro* and *in vivo* experimental validation. DNA polymerase kappa (POLK) and mutant p53 emerged as the top-ranked candidate targets. In the studied mutant p53 PDAC model, Erav treatment significantly reduced mutant p53 protein levels and was associated with marked downregulation of POLK protein expression. POLK is a previously underexplored DNA polymerase that has been reported to be overexpressed in multiple cancer types. In a subcutaneous xenograft model, Erav treatment resulted in a 76% reduction in tumor volume. Our findings demonstrate an association between Erav treatment and reduced POLK protein expression in the studied mutant p53 PDAC model, supporting POLK as a prioritized candidate for further investigation and providing preliminary mechanistic insight into Erav activity. This integrative computational–experimental pipeline offers a robust strategy for accelerating drug repurposing in oncology.

## Graphical Abstract

**Graphical Abstract:**
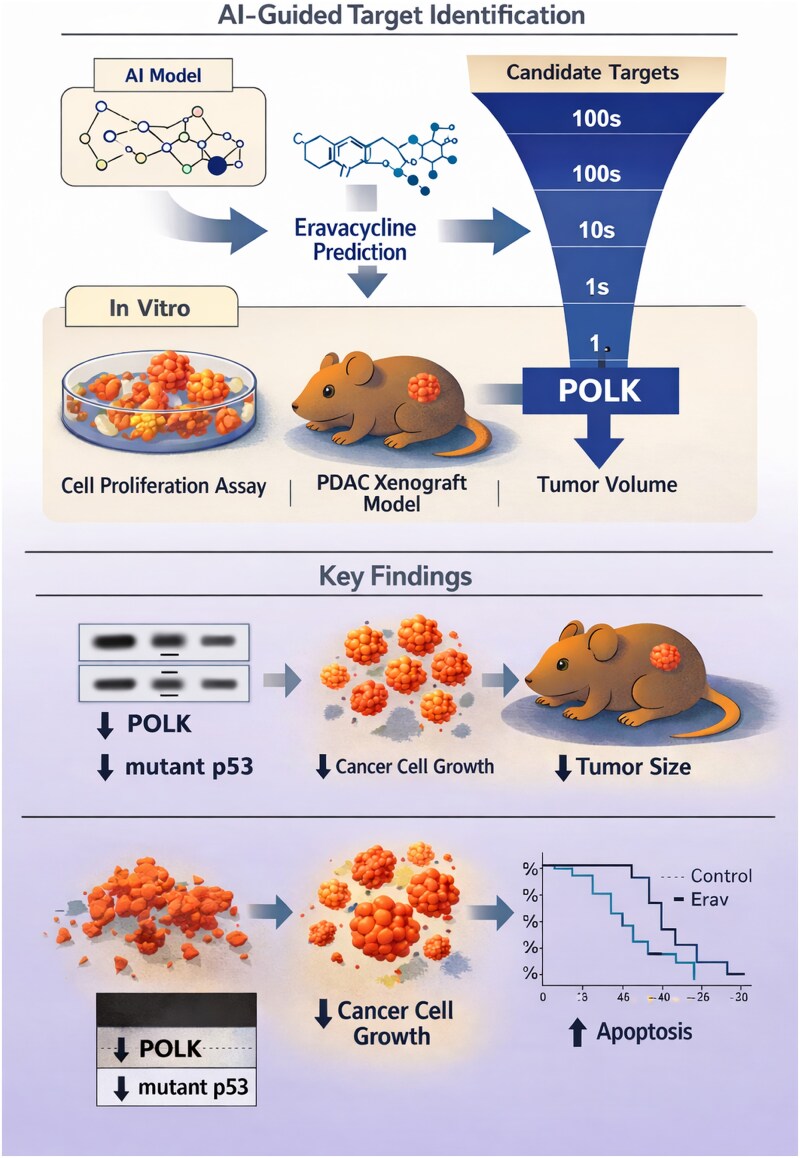

## Introduction

Pancreatic cancer remains one of the most challenging malignancies to treat, with limited effective therapeutic options, especially due to its advanced stage at diagnosis and its inherent resistance to chemotherapy [1, 2]. As one of the most lethal malignancies, it stands as the major cause of cancer-related fatalities in Western countries [3]. The intricacies of pancreatic ductal adenocarcinoma (PDAC) resistance mechanisms, marked by multiple changes in signaling pathways, contribute to its devastating nature [4]. With an average survival rate of around 5 years, conventional treatments such as chemotherapy, radiation, and surgery yield limited success, highlighting the critical need for innovative approaches [5].

Using molecular-based deep learning models to repurpose FDA-approved drugs originally intended for different indications has become widely recognized as a cost-efficient method for identifying novel anticancer therapeutics while reducing the need for extensive toxicological assessments. This strategy, which capitalizes on existing drugs, can achieve higher success rates than the conventional route of developing new chemical entities. Integrating deep learning in predicting new applications for approved drugs, particularly in diseases like pancreatic cancer characterized by ambiguous mechanisms, represents a valuable approach. Despite increased interest in repurposing drugs for PDAC, noteworthy successes remain scarce, with research often depending on hypotheses related to pharmacological mechanisms [6, 7].

While Erav, developed and approved in 2018, showed robust antibacterial activity [8], its anticancer potential remained largely unexplored. Tetracycline family drugs, known for their broad-spectrum antimicrobial properties, have also been investigated for a variety of non-antimicrobial applications, including cancer and inflammatory disorders [8, 9]. Research indicates a connection between the composition of bacterial communities and the onset of specific cancers [10]. Despite these advancements, the exact mechanisms driving this influence remain incompletely understood, necessitating continued exploration. Building on these insights, our study uses a message-passing deep neural network to predict potential targets for small-molecule drugs. Using a molecular-based deep learning model, our previous research identified Erav as a noteworthy candidate for repurposing in treating pancreatic ductal adenocarcinoma, underscoring its potential in this context [11].

Expanding upon this initial discovery, our research takes a comprehensive approach by employing a machine learning (ML) model to delve deeper into the intricacies of predicting the mechanisms of action (MOAs) associated with the anticipated anticancer drugs. To explain the MOA behind the predicted anticancer drugs and complete the virtual screening process, we developed a two-phase ML model. In the first phase, we trained an *in-silico* yeast screening model based on an extensive database with over 1.5 million molecules. This model predicted three possible outcomes: “Interaction exists,” “Confirmed non interaction,” and “Not tested.” This approach avoids the common but biologically unsupported assumption that all untested pairs are non-interactions. In the second phase, we trained a more focused binary model on the top 100 predicted interactions to refine and improve the predictions. This advanced ML model allows us to unravel the underlying biological pathways and molecular interactions contributing to the drugs’ efficacy against cancer. The analysis revealed POLK and mutant p53 as the highest-ranked targets.

Tumor protein p53 (*TP53*), which encodes the p53 protein, is crucial in tumor suppression. It is the most frequently mutated gene in human cancer, found in over 50% of pancreatic adenocarcinomas. Despite being the most frequently mutated gene, most *TP53* mutations in cancer are missense mutations, producing full-length mutant p53 protein. While the tumor-suppressing role of wild-type p53 is well-established [12], studies suggest that many mutant p53 lose this tumor-suppressing function and gain new activities that promote tumor development, indicating an oncogenic role through gain-of-function mechanisms [13].

Mutant p53 frequently accumulates to high levels in tumors, contributing to malignant progression. Given its various gain-of-function effects, researchers have identified mutant p53 as an attractive therapeutic target for cancer [14]. These effects include promoting tumor cell proliferation, survival, migration, invasion, and chemoresistance, disrupting normal tissue architecture, and altering cancer metabolism. Studies using *TP53* knock-in mouse models with mutant p53 gain of function have shown that these mice develop more aggressive or earlier tumors than *TP53* null mice [14].

POLK belongs to a family of low-fidelity DNA polymerases crucial for DNA repair, particularly in spontaneous and DNA damage-induced mutagenesis. Alterations in *POLK* gene expression have been observed in various cancer types, contributing to genome instability. Furthermore, upregulation of POLK in many cancers is associated with poor survival and reduced response to chemotherapy [15]. Therefore, identifying POLK inhibitors holds promise as potential interventions to enhance cancer treatment efficacy.

This study focuses on computational prediction and initial pharmacological validation of Erav activity in pancreatic cancer models. Detailed mechanistic experiments, including POLK knockout/rescue, pharmacokinetic profiling (plasma/tumor Erav levels), biochemical binding assays, and extensive *in vivo* mechanistic validation, are beyond the scope of the present work and are currently being pursued as part of a dedicated follow-up study.

## Materials and methods

### *In-silico* yeast screening

**Figure 1:**
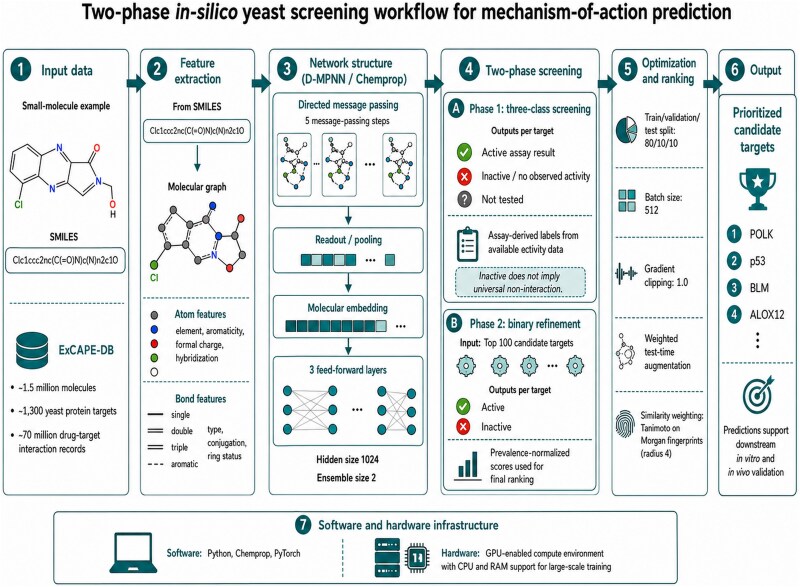
Two-phase ML approach for drug–target interaction prediction. In Phase 1, a message-passing neural network is trained on a large dataset of over 1.5 million molecules and 1300 target yeast proteins, predicting 3 outcomes: “Interaction exists,” “Confirmed non interaction,” or “Not tested.” The complementary value of the “Not tested” class is used to identify potential targets for experimental testing. In Phase 2, a focused binary per target classifier is trained on the top 100 predicted targets, refining predictions by classifying interactions as either “Interaction exists” or “Interaction does not exist.”

We trained a message-passing neural network for *in silico* yeast screening [16] to help infer potential drug mechanisms of action (Fig. 1). The model was used to train on the ExCAPE-DB database [17], which includes over 1.5 million molecules, 1300 target yeast proteins, and 70 million drug–target interactions (DTIs). The first model’s output consists of one of three outcomes for each target: “Interaction exists” (label 1), “Confirmed non interaction” (label 0), or “Not tested” (label 2). This choice explicitly forces the model to learn the difference between a confirmed non-interaction (label 0) and a lack of information (label 2). The second model, trained specifically on the top 100 targets, was a binary classifier focusing only on “Interaction exists” and “Interaction does not exist.” This approach allowed us to refine predictions and reduce model sparsity. By analyzing the complementary of “Not tested” (label 2) class, we inferred potential drug–target interactions that could be tested in the lab. This approach allows us to distinguish confirmed negatives from untested pairs. After identifying the top 100 drug–target interactions using the first model, we trained a second, more focused model. This second model was a binary classifier, designed specifically for these top 100 targets. It included two classes: “Interaction exists” and “Interaction does not exist.” This approach reduced the model’s sparsity and provided a more accurate prediction for the most promising interactions. Focusing on the top 100 targets allowed us to address the challenge of optimizing a multi-output network that operates on 1300 targets, as it is significantly easier to achieve better performance and learning efficiency with a smaller, more relevant set of targets that align with the network’s strengths. The second model used the same dataset as the first, with a train-validation-test split of 80%, 10%, and 10%, respectively.

Because ExCAPE-DB-derived predictions may not fully capture human-specific protein folding, posttranslational modifications, or cellular context, the DTI outputs should be interpreted as target-prioritization hypotheses rather than direct evidence of target engagement.

Models in both phases used the D-MPNN architecture from the Chemprop library [18–20]. Each model was trained using a batch size of 512, an ensemble size of 2, three feed-forward layers with a hidden size of 1024, a graph neural network (GNN) depth of 5, and a gradient clipping value of 1.0. Additional details about the hyperparameters used during training are provided in Supplementary Table S1.

Inspired by test-time augmentation techniques, which are beneficial for creating accurate models [21], we used the weighted average prediction of similar drugs. Our research is a collaborative endeavor between pharmacologists and computational teams, combining the strengths of both disciplines to enhance the drug discovery process. We aim to develop a general DTI prediction model and use it to elucidate why Erav exhibits anticancer properties. This process combines several steps, including binary modeling, DTI modeling, and both *in vitro* and *in vivo* experiments. Understanding the importance of predicting DTI at the right point in this process is crucial for achieving accurate and meaningful results. Here, we focused on Erav, a tetracycline-family compound. For the test-time augmentation step, we used ERV together with six tetracycline-family analogs annotated as having reported anticancer activity: tigecycline, minocycline, doxycycline, tetracycline, metacycline, and incyclinide/COL-3. These compounds were selected using two predefined criteria: structural membership in the tetracycline scaffold family and prior reported anticancer relevance. The goal was not to include every tetracycline-like compound available in the dataset, but to restrict the weighting step to structurally related compounds with anticancer annotation. The weights were calculated using Tanimoto similarity over Morgan fingerprints with radius 4. To set the classification thresholds for each target, we calculated a weighted prediction score normalizing the model’s prediction by the prevalence of each target.

To benchmark our model’s ability to identify relevant targets, we compared our findings against two established DTI prediction tools, SSM-DTA [22] and DeepDTAGen [23]. We used this benchmark to predict the IC50 values for Erav against our two top-ranked targets of interest: POLK (a top-ranked predicted candidate identified by our model) and p53 (a known positive control).

The SSM-DTA model [22] predicted an IC50 of 2.93 μM for POLK and a significantly weaker IC50 of 16.53 μM for p53.

Similarly, the DeepDTAGen model [23] predicted an IC50 of 5.75 μM for POLK and a weaker IC50 of 6.22 μM for p53. Within the context of ExCAPE-DB standards, where a target is defined as active if the IC50 is <5 μM, DeepDTAGen effectively classified both targets as inactive, although the predictions were near the threshold.

This comparison highlights the distinct advantage of our two-phase screening approach. While general-purpose DTI regression models like SSM-DTA and DeepDTAGen struggled to consistently identify both targets (with SSM-DTA missing the control and DeepDTAGen falling below the activity threshold for both), our model correctly prioritized both POLK and p53 as high-probability targets. This demonstrates that our method is more reliable for retrieving experimentally validated targets from a highly sparse search space.

### Cell culturing

All cell lines were obtained from ATCC. The cell lines were cultured in DMEM (HPNE) or RPMI-1640 (BxPC-3) media. Media contained 10% FBS, 1% L glutamine, and 1% penicillin–streptomycin, and cells were maintained at 37°C in a 5% CO2 humidified incubator.

### Tetracycline derivative treatment

Erav dihydrochloride [MedChem Express (MCE)] was dissolved in deuterium depleted water (DDW). The drugs were prepared as 100 mM stock solutions. Cell lines were treated with Erav (at the indicated concentrations) for 72 hours.

### Protein extraction from BxPC-3 cells

Following 72 hours incubation of BxPC-3 cells with medium alone as a control or medium containing different concentrations of Erav, Gemcitabine (GEM), or Doxorubicin (DOX), BxPC-3 cells were harvested and lysed. BxPC-3 cells were lysed in a buffer consisting of HEPES, NaCl, EGTA, EDTA, glycerol, MgCl2, Triton X-100, and protease phosphatase inhibitors. After incubation and sonication, lysates underwent centrifugation, yielding supernatants for protein concentration analysis via the Bradford assay at 595 nm using a Bio-Rad iMark Microplate Absorbance reader.

### Western blot analysis

The expression of p53 and POLK was detected using western blot assays. Actin was used as a protein loading control. After 3 days of incubation of the different pancreatic cells with medium as a control or medium containing different concentrations of Erav or GEM, cells were harvested, lysed, and whole cell protein was extracted. Then, 20 μg protein of each sample was mixed with reducing Laemmli sample buffer 1:3 (Bio-Rad, Hercules, CA, USA) containing 0.1% β-mercaptoethanol, boiled for 5 minutes at 95°C, and separated on a 10% SDS-PAGE gel. Proteins were transferred to a nitrocellulose membrane, blocked in TBS + 5% BSA with 0.5% Tween 20, and then probed overnight with primary antibodies - anti p53 and anti-POLK (1:1000, MA512557, Thermo Fisher Scientific; 1:1000, 14455-1-AP, Proteintech, respectively). Membranes were incubated for 1 hour at room temperature with anti-rabbit secondary antibodies (1:3000, 111-035-144, Jackson ImmunoResearch Laboratories). β-actin levels were determined using anti-β-actin (1:10 000, A3854, Sigma-Aldrich). Imaging was performed using chemiluminescence reagent (Immobilon® Crescendo Western HRP Substrate, Merck) with ImageQuant LAS 500 (GE Healthcare Bio-Sciences AB, Sweden). Semiquantitative analysis was performed for all western blot experiments using a computerized image analysis system (Mac Biophotonics ImageJ software version 1.53k14).

### Preparation and processing of BxPC-3 cell xenograft tumor model

Six-week-old female athymic nude mice were purchased from Envigo (Jerusalem, Israel), housed in pathogen-free conditions, and fed a standard sterilized diet. The mice were housed in humidity- and temperature-controlled conditions, and the light/dark cycle was set at 12 hours intervals. The Committee for the Ethical Care and Use of Animals in Research reviewed and approved the animal protocol at Ben-Gurion University of the Negev, complying with the Israeli Animal Welfare Law. BxPC-3 cells (4 × 10^6^) were injected subcutaneously into one flank of the mice. Tumor size was measured every other day based on caliper measurements of tumor length and width (volume = tumor length × width^2^ × 0.5236). Ten days later, when tumors were palpable and reached 50–150 mm^3^ volumes, the mice bearing tumors were randomly assigned to control, GEM, or Erav groups (*n* = 6). Treatment was administered every 2 days using either phosphate-buffered saline (PBS), Erav (10 mg/kg by i.p. injection), or GEM (25 mg/kg by i.p. injection) for 10 days. The tumor growth and mouse weight were measured every other day during treatment. After the last treatment, the mice were monitored for 6 more days, and the experiment was terminated. Mice were then sacrificed; the tumors were excised, photographed, and measured, and the tumor tissues were snap-frozen pending analysis.

Statistical analysis of *in vivo* data was performed using one-way analysis of variance (ANOVA) followed by *post-hoc* multiple comparison testing where appropriate. Tumor volume and body weight data are presented as mean ± SD. A *P*-value <.05 was considered statistically significant. Effect size estimates were calculated to aid interpretation of treatment effects in this proof-of-concept study.

## Results

### *In-silico* yeast screening

For the *in-silico* yeast screening model, in Table 1, we report an average area under the receiver operating characteristic curve (AUC) of 0.939 and area under the precision-recall curve (AUPR) of 0.99 for the task of identifying the absence of an interaction (note that the inverse of this class attribute can be used to identify the presence of any interaction); an AUC of 0.913 and an AUPR of 0.178 for identifying activation of a target; and an AUC of 0.95 and AUPR of 0.19 for identifying inactivation of a target.

**Table 1:** Results for *in-silico* yeast screening, first model’s three-class classification task trained using a D-MPNN model on ExCAPE-DB including 1300 targets.

**Outcome**	**AUPR**	**AUC**	**Prior probability of class in dataset**
Absence of an interaction	0.990	0.939	96.4%
Activation of a target	0.178	0.913	0.7%
Inactivation of a target	0.190	0.950	3.9%
**Average**	0.453	0.934	

**Figure 2:**
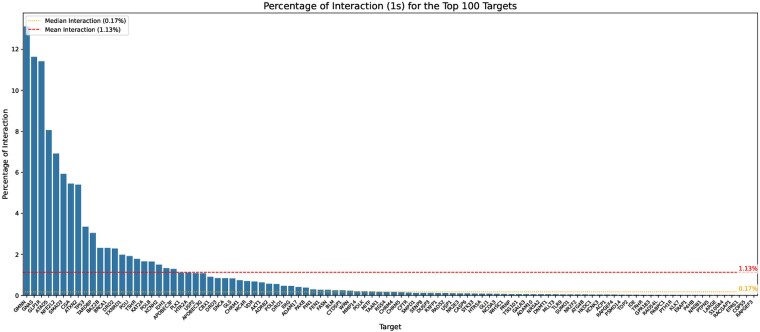
Data distribution of the top 100 targets in ExCAPE-DB. Bars represent the percentage of interaction for that specific target only (number of 1 s divided by the count of the target in the database). The upper dashed line indicates the mean interaction percentage in the entire database, whereas the lower dashed line indicates the median interaction percentage.

The difference between high AUC and low AUPR is an expected outcome in highly imbalanced datasets. AUPR is highly sensitive to the prevalence of the positive class. As shown in Fig. 2, the ExCAPE-DB dataset is extremely skewed, with a mean interaction prevalence of only 1.13% and a median of 0.17% on the top 100 targets.

Therefore, our model’s AUPR scores (0.178 and 0.19), while numerically low, represent a substantial improvement over this baseline and support the model’s utility for rank-based target prioritization. Because the first phase is a highly imbalanced three-class screening model used to select candidate targets rather than a calibrated fixed-threshold classifier, threshold-dependent metrics such as precision and recall are less informative at this stage and depend strongly on the chosen operating threshold. Accordingly, Phase 1 performance is reported using AUC/AUPR, while threshold-dependent metrics are reported for the Phase 2 binary refinement model, where such metrics are more directly interpretable.

**Figure 3:**
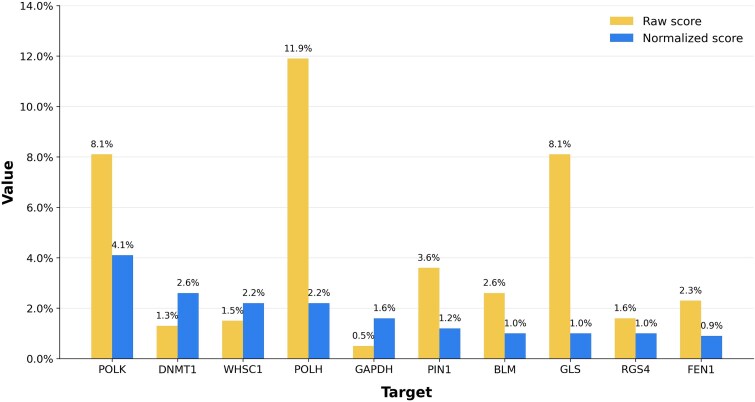
Top predicted targets by the Phase 2 binary refinement model, showing raw and normalized prioritization scores. The normalized score is calculated by dividing the raw prediction score by the prior probability of each target, then scaled down by a factor of 1000 for clarity. These scores represent the final prioritization outputs used for downstream target selection.

To identify candidate target proteins that may explain Erav’s predicted anticancer activity, we manually analyzed the top 100 predicted targets for Erav, which were identified using the complement of the “Not tested” class from the first model. Among the top predicted interactions for Erav by the first model, CYP3A4 ranks fifth. This aligns with information from DrugBank (drugbank.com), which states that Erav is primarily metabolized by CYP3A4. This external confirmation adds confidence to the model’s predictions, particularly as Erav was not part of the training set. We report additional targets identified by analyzing the first model’s output for Erav and focusing on targets known to take part in anticancer MOAs. The targets are p53, ALOX12, POLK, and BLM. The only target that was not associated with PDAC is POLK. While the first model provided an overall map of potential interactions, the second model refined these predictions by focusing on the most promising targets. A second model was trained on these top 100 targets to improve the accuracy of predicting interactions. Table 2 shows the results of the binary model. The model showed strong performance, with an AUC of 0.78 ± 0.14, an AUPR of 0.29 ± 0.13, and an F1-score of 0.25 ± 0.11. Similar to the first model, AUPR reflects the class imbalance, yet it shows significant improvement above a random baseline. To further assess the stability of the Phase 2 selected target predictions, we calculated 95% confidence intervals (CIs) for the Erav target probabilities. This was done by applying a nonparametric bootstrap method across the five-fold CV prediction results [24]. The 95% CI for the predicted interaction probability for POLK was (0.0280, 0.1999), and for p53, it was (0.0117, 0.0678). These relatively wide intervals transparently reflect the prediction variance observed across folds, partly driven by high variance in one fold, and provide an estimate of prediction stability. Figure 3 shows a bar chart illustrating the top 10 targets predicted by the second binary classifier. The yellow bars represent the raw prediction score, while the blue bars depict the normalized score, which adjusts for target prevalence in the dataset. POLH, POLK, and GLS show the highest raw prediction scores, while POLK, GAPDH, and BLM have significant normalized scores, highlighting their potential relevance as drug targets.

**Table 2:** Results for *in-silico* yeast screening trained using a D-MPNN model on ExCAPE-DB including the top 100 targets for Erav. Dataset size: Train = 781 284, Validation = 97 660, Test = 97 661. Number of folds: 5.

**Metric**	**Value**
AUC	0.78 ± 0.14
AUPR	0.30 ± 0.13
Accuracy	0.88 ± 0.18
F1 score	0.25 ± 0.11
MCC	0.28 ± 0.14
Recall	0.29 ± 0.09
Precision	0.53 ± 0.25
Binary cross-entropy	0.19 ± 0.24

### Eravacycline reduces PDAC cell survival and alters protein expression

Our previous study showed that Erav has a potent anticancer activity [11], particularly against pancreatic cancer cell lines (BxPC-3 and AsPC-1), while showing limited efficacy in other cancer cell types (MCF7, HT-29, and A-549).

Our previous paper also showed that after treatment of PDAC cells with Erav, the process of apoptotic cell death was activated. By applying a two-phase ML approach, we first identified potential interactions by ranking targets using the probability from our three-class model. In the second phase, a binary model was trained on the top 100 targets, leading to significantly improved accuracy in identifying targets likely to interact with Erav.

**Figure 4:**
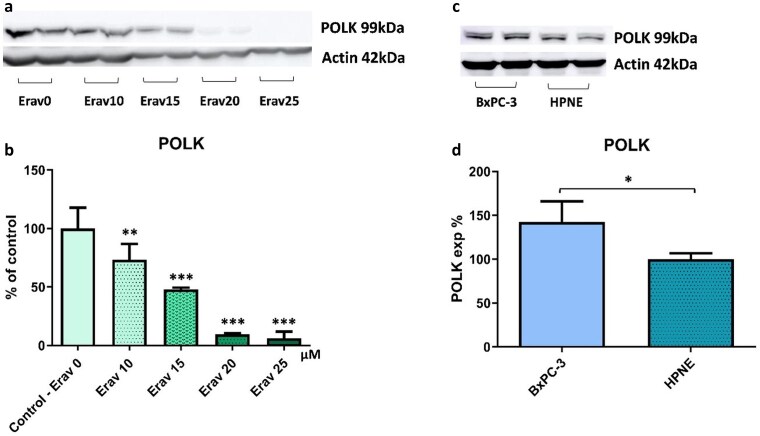
Eravacycline reduced POLK expression in BxPC-3 cells. (a, b) BxPC-3 cells after treatment with increasing concentrations of Erav (0, 10, 15, 20, and 25 μM) for 72 hours. The expression of POLK-related protein was detected using western blot assay. (c, d) Comparison of baseline POLK protein expression in pancreatic cancer cells (BxPC-3 cell line) versus pancreatic normal cells (HPNE cell line) using western blot analysis; actin was used as control. At least three independent experiments were conducted. All data are shown as the mean ± SD. A comparison between two groups was performed using a student’s *t-*test, and comparisons between multiple groups were performed using a one-way ANOVA. ^*^P < .05; ^**^P < .01, ^***^P < .001. ns, not significant. *P*-value <.05 was considered statistically significant.

**Figure 5:**
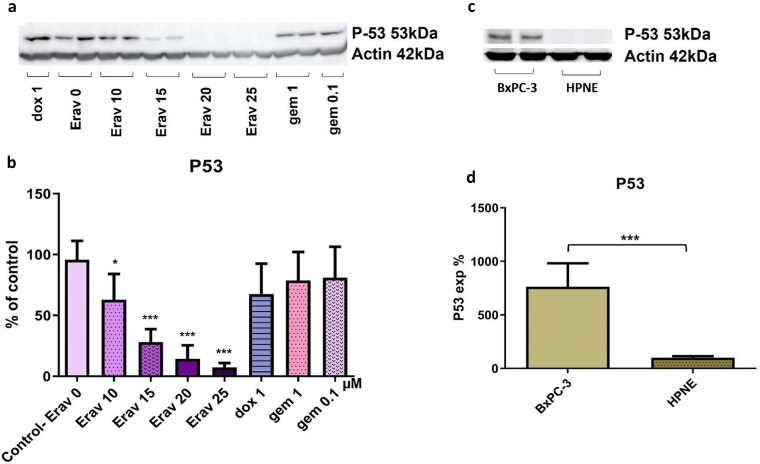
Eravacycline suppresses the expression of p53 protein in BxPC-3 cells. (a, b) cells were treated with various concentrations of Erav (0, 10, 15, 20, and 25 μM), DOX (1 μM), or GEM (0.1 and 1 μM) for 72 hours. (c, d) Comparison of baseline p53 protein expression between pancreatic cancer cells (BxPC-3 cell line) with mutant p53 and pancreatic normal cells (HPNE cell line) with wild-type p53 using western blot analysis. The expression of p53 protein was detected using western blot assay; actin was used as control. All data are shown as the mean ± SD. A comparison between multiple groups was performed using a one-way ANOVA. ^*^P < .05; ^***^P < .001. ns, not significant. *P*-value <.05 was considered statistically significant.

To further investigate the biological effects associated with Erav treatment in BxPC-3 cells, we examined the expression of POLK, the top-ranked candidate target identified by the second model, using western blot analysis. We also included p53 in our investigation as it was identified by the first model as a high-ranking target and because of its established role in PDAC pathogenesis, its involvement in critical pathways governing cell cycle regulation and apoptosis. *TP53*’s frequent dysregulation in PDAC provides a complementary pathway for evaluating the broader therapeutic effects of Erav. Our results demonstrated that Erav treatment significantly decreased the expression of p53 and POLK (Figs 4 and 5). It is important to note that changes in POLK and p53 levels reflect modulation of expression and do not, by themselves, demonstrate direct biochemical inhibition.

Figure 4 presents the results of our experiment on the effect of increasing concentrations of Erav for 72 hours on the expression of POLK in BxPC-3 cells. We found the expression of POLK was significantly reduced in protein expression levels (Fig. 4a and b) after being treated with Erav in a dose-dependent manner compared to the control group. Our experiments also show that POLK baseline expression levels were significantly higher (Fig. 4c and d) in pancreatic cancer cells (BxPC-3 cell line) compared to pancreatic normal cells (HPNE cell line).

### Eravacycline treatment is associated with reduced mutant p53 protein levels in PDAC cells

Figure 5a and b present the results of our experiment on the effect of increasing concentrations of Erav for 72 hours on the expression of p53 in BxPC-3 cells. Cells were treated with various concentrations of Erav (0, 10, 15, 20, and 25 μM), DOX (1 μM), or GEM (0.1 and 1 μM), known anticancer agents. Cells were harvested, and protein extracts were analyzed by western blotting to assess the levels of p53. The results indicate a dose-dependent reduction in p53 expression with increasing concentrations of Erav, as well as a comparative analysis of the effects of DOX and GEM on p53 expression. Moreover, our experiments notably revealed a significant difference in baseline p53 expression levels between pancreatic cancer cells (BxPC-3 cell line) with mutant tp53 and pancreatic normal cells (HPNE cell line) with wild-type tp53 (Fig. 5c and d).

### The effect of eravacycline on the growth of PDAC xenografts

**Figure 6:**
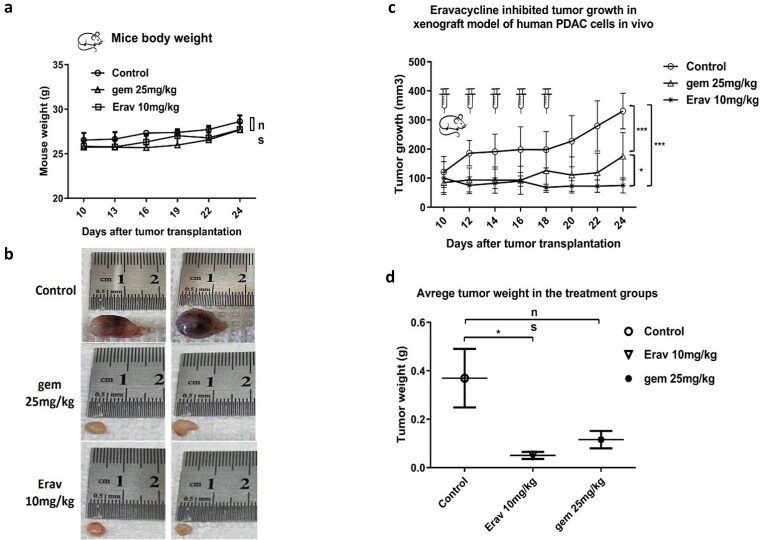
Eravacycline inhibits tumor growth in xenograft model of human PDAC cells *in vivo*. (a) Mouse weight averages for each group. (b) Photograph of tumors resected from mice in each group. (c) The percentage of change in the xenograft tumor size in the mice groups: the control group, the GEM group (25 mg/kg), and the Erav group (10 mg/kg). (d) Tumor weight average for each group. Two independent experiments were conducted. All data are shown as the mean ± SD. A comparison between multiple groups were performed using a one-way ANOVA. ^*^P < .05; ^***^P < .001. *P*-value <.05 was considered statistically significant.

To further assess the inhibition effect of Erav on human PDAC cells *in vivo*, BxPC-3 cells were injected subcutaneously into nude mice to establish xenograft models for *in vivo* experiments. Then, the mice were randomly divided into the control group, the GEM group (25 mg/kg), and the Erav group (10 mg/kg). Following the last treatment administration, mice were monitored for an additional 6 days. The experiment was terminated, the mice were sacrificed, and the tumors were excised, photographed, and weighed (Fig. 6). The results revealed that Erav treatment significantly suppressed tumor growth by 76% (Fig. 6c). No statistically significant differences in body weight were observed between the Erav-treated and control groups throughout the treatment period (one-way ANOVA, *P* > .05) (Fig. 6a). These data show that Erav effectively inhibited the development of tumors *in vivo*.

## Discussion

Using two ML models enabled us to prioritize candidate drug–target associations and generate hypotheses regarding Erav-associated anticancer activity through *in silico* yeast screening based on an extensive database of more than 1.5 million molecules.

Potential mechanisms of action of the approved drugs were investigated using a two-phase ML approach. The first phase used a message-passing neural network trained on ExCAPE-DB [17], which consists of 1.5 million molecules and 1300 yeast targets, to systematically predict interactions based on 3 classes: “Interaction exists,” “Confirmed non-interaction,” and “Not tested.” The second phase focused on the top 100 targets identified from the first model, using a binary classifier to refine predictions and improve accuracy in identifying the most promising interactions. By narrowing the focus to the top 100 targets, the second model allowed for a more detailed and accurate assessment of drug–target interactions, reducing the computational complexity while improving precision in the predictions. Notably, neither data oversampling nor synthesis techniques were employed in the training of either model.

For the *in-silico* yeast screening model, we report an average AUC of 0.939 and AUPR of 0.99 for identifying the absence of interaction, an AUC of 0.913 and AUPR of 0.178 for identifying the activation of a target, and an AUC of 0.95 and AUPR of 0.19 for identifying the deactivation of a target. These AUPR values represent 25-fold and 4.9-fold improvements over a random classifier. These values indicate that the model can prioritize candidate interaction signals in this highly imbalanced screening setting, supporting its use as a hypothesis-generation step rather than as definitive evidence of an anticancer mechanism.

While predicting mammalian targets from the yeast-based ExCAPE-DB dataset carries inherent translational challenges, this approach is firmly established in computational drug discovery, with ExCAPE-DB routinely utilized as a predictive oracle for human targets within multi-objective molecular optimization frameworks [25–28]. Because the ExCAPE-DB-based model is used here for target prioritization rather than direct mechanistic inference, its predictions should be interpreted as hypothesis-generating and require downstream validation in mammalian systems.

Discovered in the late 1940s, tetracyclines are broad-spectrum antibiotics used to this day to treat infectious diseases. Tetracyclines’ antimicrobial effect is mediated by the inhibition of protein translation through binding to the bacterial 30S ribosomal subunit; this blocks the attachment of aminoacyl-tRNA to the A-site of the ribosome, thus preventing protein synthesis [29]. Interestingly, previous studies have shown that tetracyclic analogs induce apoptosis in several cancer cell lines (prostate, breast, gastric cancer, leukemia, melanoma, osteosarcoma, colorectal, and pancreatic cancer) [6, 29–31]. One study investigating the cytotoxic potential of tetracyclic analogs showed that doxycycline, minocycline, and chemically modified tetracycline-3 (COL-3) have therapeutic potential in cancer treatment [29]. Moreover, recent studies suggested that tigecycline, a new member of the tetracycline class of antibiotics that is active against gram-negative and gram-positive bacteria (which are particularly drug-resistant pathogens) [32], may be used as an adjunct to treatment in various types of cancer [31].

The modes of action of anticancer tetracycline drugs are diverse. Doxycycline and minocycline are known for inducing apoptosis in amelanotic melanoma cells. Treatment with these compounds led to modifications in the cell cycle and decreased the intracellular level of reduced thiols and mitochondrial membrane potential. The anti-melanoma effect was related to the upregulation of the target ERK1/2 and MITF [30]. Tigecycline has been shown to have possible anticancer activity and exert its action in PDAC via the downregulation of CCNE2 [6]. Another study showed that tigecycline inhibited mitochondrial respiration and translation in thyroid carcinoma cells [33]. A study that investigated the cytotoxic effects of tetracyclic analogs (doxycycline, minocycline, and chemically modified tetracycline-3) found that apoptosis is induced via mitochondria-mediated and caspase-dependent pathways in the human myeloid leukemia cell line [29] by inhibiting matrix metalloproteinases (MMPs) and directly affecting cell proliferation [34, 35].

To explore potential protein targets associated with Erav treatment and gain initial insight into its possible mode of action, we applied an *in-silico* yeast screening framework to prioritize candidate targets. Model predictions yielded multiple potential targets. We ranked these candidates based on the yeast screening model’s predicted probability scores to identify the highest-confidence targets for further analysis. We then manually analyzed the top 100 targets predicted for Erav by comparing the model’s prediction for each target with a target-specific threshold proportional to the number of positive cases in the training set. We focused our analysis on targets with existing evidence of anticancer activity.

Our analysis of the model’s predictions indicated that p53, ALOX12, BLM, and POLK were among the top-ranked candidate targets associated with Erav’s predicted anticancer activity. The expression of p53, ALOX12, and BLM has been associated with PDAC [36], while the involvement of POLK in PDAC remains unexplored. In the experiment, after being treated with Erav in a dose-dependent manner, we found that the expression of POLK was significantly decreased (Fig. 4). POLK is a member of a family of low-fidelity DNA polymerases involved in DNA repair implicated in spontaneous and DNA damage-induced mutagenesis [15].

POLK expression has been reported to be dysregulated across multiple cancer types, and its upregulation has been associated with genomic instability, poor clinical outcome, and reduced sensitivity to DNA-damaging agents [15]. Prior studies have suggested that POLK may represent a potential therapeutic vulnerability in certain malignancies, and that modulation of its activity could enhance treatment response [37]. In our study, reduced POLK protein expression was observed following Erav treatment, and this change may be associated with the compound’s antiproliferative activity in pancreatic cancer. However, the mechanistic significance of POLK expression changes in PDAC remains incompletely defined.

POLK is a member of the Y-family of low-fidelity DNA polymerases and participates in translesion DNA synthesis (TLS), a DNA damage tolerance pathway that enables replication across damaged templates. TLS involves multiple specialized polymerases, including POLH, POLI, REV1, and the extender polymerase REV3L, each contributing distinct lesion-bypass functions. More broadly, DNA polymerases influence tumorigenesis in different ways depending on their functional class. Replicative polymerases such as POLD1 and POLE have been implicated as driver events in certain cancers through proofreading defects that promote hypermutation phenotypes [38].

In contrast, TLS and other reparative polymerases are more frequently linked to DNA damage tolerance and therapy resistance rather than direct tumor initiation. Notably, several TLS polymerases—including POLK—have been reported to be upregulated in PDAC relative to normal pancreatic tissue [38]. It therefore remains unclear whether elevated POLK expression in PDAC represents a functional driver event contributing to tumor progression or a passenger adaptation reflecting replicative stress and underlying genomic instability. Our data demonstrate reduced POLK protein expression following Erav treatment but do not establish a direct mechanistic role or confirm target engagement. The observed reduction should be interpreted as a preliminary mechanistic clue rather than definitive evidence of biochemical interaction or direct target engagement. Accordingly, the present findings are best viewed as identifying a phenotypic association and a prioritized candidate target rather than establishing a direct molecular mechanism of ERAV action.

Based on POLK’s role in TLS, one possibility is that reduced POLK expression could influence the capacity of PDAC cells to tolerate endogenous or therapy-induced DNA damage. Altered DNA damage tolerance may, in turn, affect genomic stability and apoptotic signaling. However, this proposed mechanism remains speculative and was not directly evaluated in the current study [15]. Functional perturbation studies, including POLK knockdown or rescue approaches, will be required to determine its relative contribution compared with other DNA repair polymerases and to clarify its therapeutic relevance in PDAC.

Previous work demonstrated that Erav exhibits antiproliferative activity in pancreatic cancer models, with IC50 values of 3.57 μM in BxPC-3 cells and 7.07 μM in AsPC-1 cells. In addition, Erav was evaluated across multiple cancer cell lines, including breast (MCF-7), lung (A549), and colon (HT-29), where differential sensitivity was observed. These findings provide important context for the anticancer activity of Erav and support its potential as a repurposed therapeutic candidate [11].

We examined the effect of Erav on p53 protein expression, as TP53 was identified by the first computational model as a top-ranked predicted target. In addition, TP53 plays a well-established role in PDAC pathogenesis, with mutations occurring frequently in pancreatic cancer and significantly influencing cell cycle regulation and apoptotic pathways. In our experiments, we observed a significant reduction in mutant p53 protein levels in the BxPC-3 cell line following Erav treatment, with dose- and time-dependent effects (Fig. 5). This reduction may be associated with alterations in oncogenic pathways linked to p53 dysfunction in PDAC. Consistent with previous reports, mutant p53 accumulation in tumors has been shown to contribute to gain-of-function activities that promote tumor progression and are often associated with more advanced disease [39]. In addition to our findings, previous studies suggest that mutant p53 protein accumulation contributes to tumor progression and gain-of-function oncogenic activity. Whether the reduction in mutant p53 levels observed following Erav treatment directly contributes to the inhibition of cancer cell proliferation remains to be determined. The mechanisms underlying mutant p53 accumulation in tumors are not fully understood, and improved insight into these processes may inform future therapeutic strategies targeting mutant p53-driven cancers. Our *in vivo* studies showed that human pancreatic carcinoma cells (BxPC-3) were responsive to the growth-inhibitory effects of Erav (76% inhibition at 10 mg/kg) compared with GEM (45% inhibition at 25 mg/kg). In a xenograft model using female athymic nude mice, Erav treatment was associated with significant tumor growth inhibition.

These findings suggest that Erav may have potential as a less toxic complementary or alternative agent to GEM. While GEM remains a well-established standard chemotherapy, the observed activity of Erav at lower concentrations indicates that further investigation is warranted to better understand its possible therapeutic role in pancreatic cancer. The *in vivo* study was conducted using *n* = 6 mice per group, a sample size commonly employed in exploratory xenograft studies aimed at pharmacological proof-of-concept. While not powered for definitive efficacy conclusions, the observed effect sizes and consistent tumor growth inhibition provide a rationale for future, larger-scale studies incorporating formal power calculations.

The present study has several limitations. First, although the deep learning framework enabled prioritization of candidate targets, model-derived predictions are inherently probabilistic and require further validation, particularly given the translational challenges associated with extrapolating from yeast-based datasets to human biological systems. Second, while reduced POLK and mutant p53 protein expression was observed following Erav treatment, direct biochemical evidence of target engagement was not evaluated, and the observed effects may reflect indirect regulatory mechanisms. Third, all *in vitro* experiments were conducted exclusively in the BxPC-3 cell line, which harbors mutant TP53. Therefore, our *in vitro* conclusions are presently restricted to the mutant p53 context of the BxPC-3 model. Given the known influence of TP53 status on cellular responses to stress and therapeutic agents, additional validation in PDAC cell lines with wild-type TP53 and diverse genetic backgrounds will be required to assess the broader applicability of these findings. Fourth, protein expression was assessed at a single time point and within a limited concentration range, and therefore, the dose–response relationship and temporal dynamics of POLK and mutant p53 modulation were not fully characterized. Fifth, although POLK downregulation was associated with antiproliferative effects, the present study does not directly assess downstream functional consequences such as DNA damage accumulation or replication stress, and thus, a causal link between POLK modulation and the observed phenotype remains to be established. Finally, the subcutaneous xenograft model used in this study, while widely applied, does not fully recapitulate the complex tumor microenvironment of pancreatic cancer, including stromal and immune interactions. Future studies using orthotopic models may provide a more physiologically relevant evaluation of Erav’s *in vivo* effects.

Beyond the specific molecular findings presented here, the present study contributes to the growing recognition of functional overlaps between antimicrobial agents and anticancer therapeutics. Several classes of antibiotics, including tetracycline derivatives, have been reported to exhibit anticancer activity through mechanisms such as mitochondrial translation inhibition, modulation of cellular stress responses, and potential effects on DNA damage tolerance pathways. Erav, as a synthetic tetracycline analog, may therefore represent part of a broader class of antimicrobial agents with potential oncologic relevance.

Evaluation of additional tetracycline derivatives and structurally related antimicrobial compounds may help determine whether the observed molecular signatures are compound-specific or reflect a broader class-related effect. Furthermore, extending this artificial intelligence (AI)-guided prioritization framework to other cancer types characterized by dysregulated DNA repair pathways may help assess the versatility and generalizability of the approach. Such cross-disciplinary integration between antimicrobial pharmacology and oncology may facilitate rational drug repurposing strategies.

In this context, the potential repurposing of Erav for oncology applications also warrants consideration of key translational aspects. As an FDA-approved antibacterial agent, Erav has an established safety profile in humans, supported by clinical studies evaluating its efficacy and tolerability in infectious disease settings. For example, a recent systematic review and network meta-analysis indicates that Erav is generally well tolerated and may be associated with fewer discontinuations due to adverse events compared to certain alternative antimicrobial agents [40]. This established clinical safety profile may facilitate its repositioning in oncology compared to *de novo* drug development. However, the translation of Erav to cancer therapy will require careful evaluation of dosing strategies, as the pharmacological requirements for anticancer efficacy may differ from those used in antimicrobial indications. In addition, potential drug–drug interactions and toxicity profiles will need to be assessed in the context of cancer patients, who often receive combination therapies. These considerations highlight the importance of further preclinical and clinical studies to determine the feasibility and safety of Erav as a repurposed anticancer agent.

## Conclusions

The AI-based deep learning framework enabled the identification and prioritization of potential molecular targets associated with Erav activity in PDAC. Integration of computational predictions with experimental validation revealed reduced POLK and mutant p53 protein expression following treatment, providing preliminary mechanistic clues rather than definitive evidence of direct target engagement.

Within the two-phase ML approach, POLK was ranked as the top predicted target in Phase 2 based on a normalized scoring system that integrated model probability and prior target prevalence, followed by DNMT1, WHSC1, POLH, and GAPDH. These findings highlight POLK as a high-ranking candidate associated with Erav treatment and warrant further functional and biochemical investigation to clarify its potential contribution relative to other DNA repair pathways.

Overall, this integrative computational–experimental strategy demonstrates the value of AI-guided approaches in generating testable hypotheses and accelerating drug repurposing efforts in oncology.

Our code: https://github.com/eyalmazuz/DrugRepurposing.

**Key Points**

- **A novel two-phase deep learning model** was developed to predict and prioritize potential molecular targets associated with the anticancer activity of repurposed drugs using an *in-silico* yeast-based screening approach.
- **Eravacycline**, an FDA-approved antibacterial agent, was computationally prioritized and demonstrated anticancer activity in preclinical models of pancreatic ductal adenocarcinoma (PDAC).
- **POLK (DNA Polymerase Kappa)** emerged as a top-ranked predicted candidate in PDAC, and ERAV treatment was associated with reduced POLK protein expression in the studied model.
- **Integration of AI-based prediction and experimental validation** showed that Erav treatment reduced mutant p53 and POLK protein levels and was associated with significant tumor growth inhibition (76%) in a xenograft model.
- This study highlights the potential of **target-based deep learning models** to accelerate oncology drug repurposing and generate testable hypotheses for further mechanistic investigation.

## Supplementary Material

Supplementary_Information_bbag353

## Data Availability

All data presented in this study are included in this published article.

## Availability and implementation

The code is available from https://github.com/eyalmazuz/DrugRepurposing.
