# Supplementary material for: Investigating the anticancer activity of eravacycline in pancreatic cancer via target-based deep learning and experimental validation
Source: Brief Bioinform. 2026 Jul 8;27(4):bbag353. doi: 10.1093/bib/bbag353 (PMC13345371; doi:10.1093/bib/bbag353)
Supplement: Supplementary_Information_bbag353 [file supplementary_information_bbag353.docx]

| Hyperparameter | Phase 1 (Multiclass) | Phase 2 (Binary Classification) |
| --- | --- | --- |
| Number of Folds | 3 | 10 |
| Ensemble Size | 1 | 2 |
| Epochs | 10 | 30 (default) |
| Batch Size | 64 | 512 |
| Message Passing Depth | 5 | 5 |
| Hidden Size | 1100 | 1024 |
| FFN Number of Layers | 2 (default) | 3 |
| Dropout | 0.1 | 0.0 (default) |
| Optimizer | Adam (default) | Adam (default) |
| Initial Learning Rate | 0.0001 (default) | 0.0001 (default) |
| Maximum Learning Rate | 0.001 (default) | 0.001 (default) |
| Final Learning Rate | 0.0001 (default) | 0.0001 (default) |
| Warmup Epochs | 2.0 (default) | 2.0 (default) |
| Gradient Clipping | 1 | 1 |

Table S1: Detailed Hyper-parameters for model training.
